# Long-term clinical outcomes of bariatric surgery in adults with severe obesity: A population-based retrospective cohort study

**DOI:** 10.1371/journal.pone.0298402

**Published:** 2024-06-06

**Authors:** Natasha Wiebe, Marcello Tonelli

**Affiliations:** 1 Department of Medicine, University of Alberta, Edmonton, Alberta, Canada; 2 Department of Medicine, University of Calgary, Calgary, Alberta, Canada; United Arab Emirates University, UNITED ARAB EMIRATES

## Abstract

**Background:**

Bariatric surgery leads to sustained weight loss in a majority of recipients, and also reduces fasting insulin levels and markers of inflammation. We described the long-term associations between bariatric surgery and clinical outcomes including 30 morbidities.

**Methods:**

We did a retrospective population-based cohort study of 304,157 adults with severe obesity, living in Alberta, Canada; 6,212 of whom had bariatric surgery. We modelled adjusted time to mortality, hospitalization, surgery and the adjusted incidence/prevalence of 30 new or ongoing morbidities after 5 years of follow-up.

**Results:**

Over a median follow-up of 4.4 years (range 1 day-22.0 years), bariatric surgery was associated with increased risk of hospitalization (HR 1.46, 95% CI 1.41,1.51) and additional surgery (HR 1.42, 95% CI 1.32,1.52) but with a decreased risk of mortality (HR 0.76, 95% CI 0.64,0.91). After 5 years (median of 9.9 years), bariatric surgery was associated with a lower risk of severe chronic kidney disease (HR 0.45, 95% CI 0.27,0.75), coronary disease (HR 0.49, 95% CI 0.33,0.72), diabetes (HR 0.51, 95% CI 0.47,0.56), inflammatory bowel disease (HR 0.55, 95% CI 0.37,0.83), hypertension (HR 0.70, 95% CI 0.66,0.75), chronic pulmonary disease (HR 0.75, 95% CI 0.66,0.86), asthma (HR 0.79, 95% 0.65,0.96), cancer (HR 0.79, 95% CI 0.65,0.96), and chronic heart failure (HR 0.79, 95% CI 0.64,0.96). In contrast, after 5 years, bariatric surgery was associated with an increased risk of peptic ulcer (HR 1.99, 95% CI 1.32,3.01), alcohol misuse (HR 1.55, 95% CI 1.25,1.94), frailty (HR 1.28, 95% 1.11,1.46), severe constipation (HR 1.26, 95% CI 1.07,1.49), sleep disturbance (HR 1.21, 95% CI 1.08,1.35), depression (HR 1.18, 95% CI 1.10,1.27), and chronic pain (HR 1.12, 95% CI 1.04,1.20).

**Interpretation:**

Bariatric surgery was associated with lower risks of death and certain morbidities. However, bariatric surgery was also associated with increased risk of hospitalization and additional surgery, as well as certain other morbidities. Since values and preferences for these various benefits and harms may differ between individuals, this suggests that comprehensive counselling should be offered to patients considering bariatric surgery.

## Introduction

Bariatric surgery leads to sustained weight loss in most recipients, and also reduces fasting insulin levels [1, 2] and markers of inflammation [3]. Bariatric surgery can reverse and/or lessen the severity of diabetes [4] and hypertension [5], and cardiovascular disease [6]. However, bariatric surgery is also associated with an excess risk of certain adverse outcomes, including venous thrombosis, reactive hypoglycemia [7], nephrolithiasis [8], fractures [9], depression, alcohol misuse, and suicide [10].

The current paradigm is that the benefits of bariatric surgery are mediated by reductions in fat mass. However, higher BMI and fat percentage do not correlate with excess mortality after adjustment for fasting insulin and chronic inflammation in the general population, and in fact appear protective [11, 12]. Hyperinsulinemia and chronic inflammation have also been linked to a wide range of health conditions, including cardiovascular disease, diabetes, fatty liver, sleep apnea, cancer, chronic obstructive pulmonary disorder, dementia, and autoimmune disorders [13, 14]. Given the beneficial effects of bariatric surgery on hyperinsulinemia as well as inflammatory biomarkers, it is possible that these effects contribute to the clinical benefits of bariatric surgery, which in turn may extend beyond those conditions that have been studied to date. Equally, given that bariatric surgery has a high failure rate [15] (defined as <50% excess weight loss [16]) and can cause complications from anatomical abnormalities of the gastrointestinal tract (e.g., malabsorption, nutritional deficiency, bacterial overgrowth) which can themselves lead to other health conditions (e.g., osteoporosis, peripheral neuropathy, depression), a comprehensive assessment of clinical outcomes is warranted.

The long-term risks and benefits of bariatric surgery have been incompletely studied. There is evidence in long-term (≥5 years) studies showing reductions in mortality, diabetes [17], cardiovascular disease [6] and cancer [17], and improvements in quality of life [18]. There is less long-term evidence on the risks of lung disease, sleep disturbance, depression, and other adverse events known to be associated with bariatric surgery in the short-term.

In this retrospective population-based cohort study of adults with severe obesity, we aimed to describe the long-term associations of bariatric surgery on a comprehensive panel of clinical outcomes such as mortality, hospitalization, and additional surgery, as well as the risk of 30 new or ongoing morbidities during follow-up.

## Methods

We report this retrospective population-based cohort study according to the STROBE guidelines [19]. The institutional review boards at the Universities of Alberta (Pro00053469) and Calgary (REB16-1575) approved this study and waived the requirement for participants to provide consent due to the minimal risk to the participants and the large sample size. The data did not contain individual identifying information. We did analyses between December 2, 2022 and August 8, 2023.

### Data sources and cohort

We used the Alberta Kidney Disease Network database [20–22], which incorporates data from Alberta Health (AH; the provincial health ministry) including data on registration, vital statistics, provider claims, hospitalizations and ambulatory care utilization; and from the clinical laboratories in Alberta. Despite its name, all adults registered with AH were included in the database; all Alberta residents are eligible for insurance coverage by AH and >99% participate in coverage. We used the database to assemble a cohort of adults with severe obesity who resided in Alberta, Canada. We followed participants until death, out-migration or study end (March 31, 2019), whichever was earliest.

### Bariatric surgery and severe obesity

We determined the incidence of bariatric surgery using procedure codes from provider claims (ICD-9 CCP codes; S1 Table). The date of the first procedure code was considered the index date for participants with bariatric surgery (recipients); these dates for bariatric surgery could range from April 1, 1997 to December 31, 2018, ensuring at least 90 days of follow-up after surgery. After matching on entry date (90 days within registration with AH, 18^th^ birthday or study start, whichever date was latest), the index dates for participants without bariatric surgery (non-recipients) were randomly drawn with replacement from the distribution of bariatric surgery dates in recipients. Non-recipients were drawn from the same adult population with severe obesity as the recipients.

We identified the specific type of bariatric surgery using the following claim codes: Roux-en-Y gastric bypass (56.93A), sleeve gastrectomy (56.93C), adjustable gastric band (56.93B, 56.93F), and unknown (56.93). In a sensitivity analysis, we used a definition from the Canadian Institute for Health Information (CIHI; www.cihi.ca), which identifies bariatric surgery, using hospitalization with ICD-10-CA CCI codes (1.NF.78.^^) which were converted to ICD-9-CM procedure codes (43.89, 44.39) [23]. This latter definition was similar to previous publications in bariatric surgery using administrative data [24–27]. We excluded bariatric codes if a participant was hospitalized, at the same time, for a gastrointestinal or abdominal cancer, or a perforated gastrointestinal ulcer (S1 Table).

**Fig 1:**
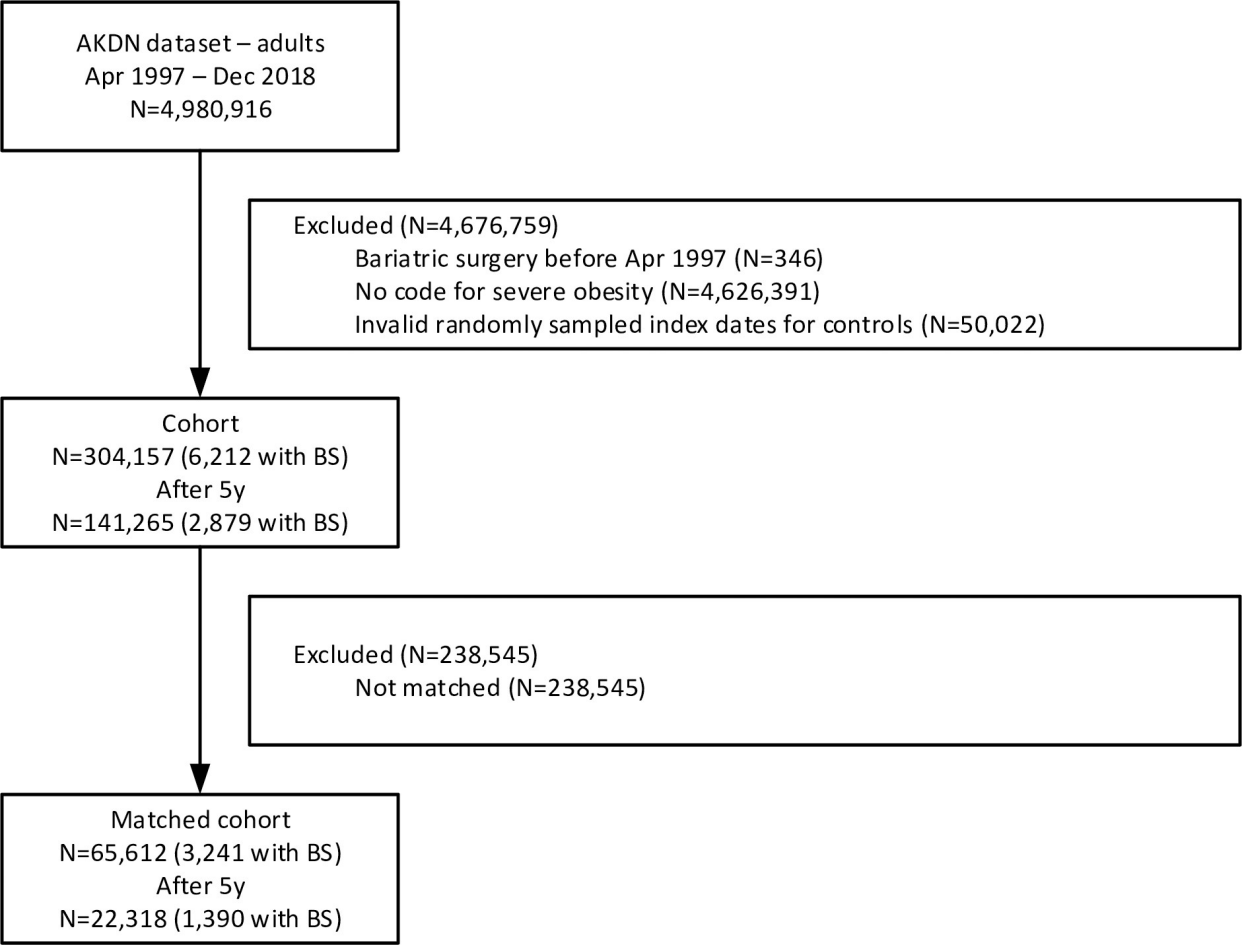
Participant flow diagram. AKDN Alberta Kidney Disease Network, BS bariatric surgery.

We considered severe obesity to be present for both recipients and non-recipients if there was at least one procedure marked with a BMI supplemental fee modifier as in our prior work [28] (≥35 kg/m^2^ before 2017 and ≥40 kg/m^2^ in/after 2017; S1 Table). From our parent dataset of registered adult Albertans, 6.1% had severe obesity and thus formed the cohort for this study (Fig 1).

Randomly sampled index dates for non-recipients were invalid if the index date occurred after their end date.

### Morbidities and other covariates

We defined morbidities using a previously published framework with validated algorithms as applied to ICD diagnosis codes (from provider claims, hospitalizations and outpatient clinics), each of which had positive predictive values ≥70% as compared to a gold standard measure such as chart review [29]. Morbidities include those potentially related to hyperinsulinemia (e.g., cardiovascular disease), inflammation (e.g., respiratory diseases), nutritional deficiencies (e.g., fragility fractures), anatomical modifications of the gastrointestinal system (e.g., peptic ulcer disease), combinations of the above, or none of the above (e.g., multiple sclerosis): alcohol misuse, asthma, atrial fibrillation, cancer (non-metastatic breast, cervical, colorectal, pulmonary, and prostate cancers, metastatic cancer, and lymphoma), chronic heart failure, chronic pain, chronic pulmonary disease, coronary artery disease (CAD; myocardial infarction, percutaneous intervention, coronary artery bypass graft), dementia, depression, diabetes, epilepsy, frailty [30] (fragility fractures: wrist, forearm, humerus, hip, pelvis, and spine; and/or osteoporosis), gout [31], hypertension, hypothyroidism, inflammatory bowel disease (IBD), irritable bowel syndrome, liver disease (chronic hepatitis B, cirrhosis), multiple sclerosis, Parkinson’s disease, peptic ulcer disease (PUD), peripheral artery disease, psoriasis, rheumatic disease, schizophrenia, severe constipation, sleep disturbance [32], and stroke or transient ischemic attack (TIA). Detailed methods for classifying comorbidity status and the specific algorithms used are found elsewhere [29]. Our dataset did not permit us to identify obstructive sleep apnea (OSA) specifically, and so we modified the previously validated algorithm to include any sleep disturbance (2 claims within 2 years: ICD-9 327, 347, and 780.5) [32]. We also considered severe chronic kidney disease (CKD) as a 30^th^ condition, which was defined by a sustained estimated glomerular filtration rate below 30 mL/min per 1.73m^2^. We classified each participant with respect to the presence or absence of these 30 chronic conditions at baseline (lookback extended as far as April 1994 where records were available) [33] and after a 5-year washout period following surgery to allow morbidities to reverse after bariatric surgery.

As in our prior work we used administrative data to identify age, biological sex, and rural residence location [34]. We included the Pampalon index of material deprivation [35, 36], which categorizes participants based on their residential postal code into 5 bins of socioeconomic inequalities in health care services and population health with 5 representing the most deprived neighbourhoods. Rural status (1.8%) and material deprivation (3.0%) were missing a few values but no other variables had missing values.

### Outcomes

Clinical outcomes were all-cause death, hospitalization, surgery, and any new or ongoing morbidities. We assessed all-cause death, hospitalization, and surgery during the first 90 days, the first 5 years (not including the index bariatric surgery but including any subsequent surgical revisions), and after 5 years until the end of follow-up. The presence of the 30 morbidities was assessed only after a 5-year washout period beginning at the time of surgery, including prevalent cases noted at the end of the washout, as well as any incident cases noted thereafter. The washout period allowed time for morbidities to reverse. Participants with dementia, multiple sclerosis, Parkinson’s disease, and schizophrenia at baseline were not included in analyses evaluating the incidence of these outcomes.

### Statistical analyses

We did analyses with Stata MP 17·0 (www.stata.com) and reported baseline descriptive statistics as counts and percentages, or means and standard deviations, as appropriate. Differences between recipients and non-recipients were tested using logistic regression.

We used unadjusted, age-adjusted and fully-adjusted Weibull hazards regression to determine the associations between bariatric surgery and time to death, hospitalization, surgery, and continuation/incidence of each comorbidity. In the fully-adjusted models, we regressed outcomes on age, biological sex, rural residence, neighbourhood material deprivation index, date of first obesity modifier, and 30 morbidities. We determined that the proportional hazards assumption was satisfied by examining plots of the log-negative-log of within-group survivorship probabilities versus log-time. We report hazard ratios with 95% confidence intervals (in text and with forest plots) and set the threshold *p* for statistical significance at 0.05.

We did several sensitivity analyses. One, in an effort to achieve strong covariate balance, we curated a cohort with (many to many) exact matches for every demographic characteristic, obesity year, and morbidity; we grouped age into 5-year bins. We also did inverse-weight propensity scoring analyses [37]. The standardized differences for the unweighted and weighted analyses are reported. Two, in the after-5-year outcomes, we modelled death as a competing risk using a flexible modelling approach outlined by Lambert [38]. We retained participants who had died in the first 5 years in this cohort; their outcome data were imputed and weighted (using the Stata command *stcrprep*) based on the conditional probability of being censored with or without bariatric surgery. Three, we regressed the number of hospitalizations, the number of days in hospital, and the number of surgeries onto bariatric surgery and all covariates using negative binomial models. Four, we parameterized follow-up duration using restricted cubic splines using 4 knots (at 1, 5, 10, and 15 years) and 6 knots (at 90 days, 1, 5, 10, 15, and 20 years). We regressed time to death on the main effects and interactions of bariatric surgery with the splines of follow-up, and further adjusted for all covariates. Five, we regressed time to deaths (over full follow-up) due to external factors (e.g., suicide and injury) and mental/behavourial disorders (ICD-10 codes starting with the letter F, X, Y, or W), and time to death due to the circulatory system (ICD-10 codes starting with the letter I). Six, we interacted age group (<40 versus ≥ 40 years) with bariatric surgery in the modelling of mortality (over full follow-up). Seven, we interacted fiscal year of the index date (as a linear term) with bariatric surgery and reported estimates for the 2000, 2005, 2010, and 2015 fiscal years. We based the 2015 estimates on extrapolations for the after-5-year outcomes. All sensitivity analyses were fully adjusted.

In order to illustrate the number of adverse outcomes averted and experienced, we calculated the numbers needed to benefit and harm using an approach by Altman and Andersen [39]. Non-recipient risk was estimated at 10 years; censoring was assumed to be non-informative. The numbers needed to benefit/harm were inverted and multiplied by 100 in order to depict the numbers of outcomes averted or experienced in a hypothetical population of 100 recipients of bariatric surgery.

## Results

### Characteristics of study participants

Participant flow is shown in Fig 1. Overall most 4,626,391 (93.8%) adult Albertans were excluded because they did not meet our definition of severe obesity. Of those with severe obesity, 6,212 (2.0%) had undergone bariatric surgery and 297,945 (98.0%) had not. The quantile-quantile plot shows that the distribution of index dates was similar for recipients and non-recipients (S1 Fig). The 304,157 participants were followed for a median of 4.4 years (range 1 day to 22.0 years). There were 15,670 deaths (5.2%); 140,121 (46.1%) participants had at least one hospitalization (for a total of 363,744 hospitalizations), and 24,165 (7.9%) had at least one additional surgery (for a total of 29,062 surgeries after the index bariatric surgery) during follow-up. For the after 5-year outcomes, the median follow-up for the 141,265 participants, with follow-up >5 years, was 9.9 years (inter-quartile range 7.2 to 14.4 years).

Table 1 summarizes demographics and clinical characteristics by receipt of bariatric surgery or not. Participants who had undergone bariatric surgery were younger, more likely to be female, and to reside in an urban residence. The morbidities were sorted by the likelihood of receiving bariatric surgery, from most to least, after adjustment for all covariates. In order of magnitude, participants were more likely to receive bariatric surgery if they had sleep disturbance, hypertension, PUD, asthma, diabetes, depression, psoriasis, hypothyroidism, chronic pain, and gout. Also, in order of magnitude, participants were less likely to receive bariatric surgery if they had severe CKD, alcohol misuse, CAD, liver disease, schizophrenia, cancer, Parkinson’s disease, dementia, heart failure, severe constipation, IBD, stroke/TIA, epilepsy, frailty, and rheumatic disease. While participants who had bariatric surgery had more morbidities on average (mean of 2.3 versus 1.8), they were more likely to have morbidities with relatively better prognosis (e.g., hypertension, depression, chronic pain) and participants without bariatric surgery were more likely to have conditions that have relatively worse prognosis (e.g., heart failure, liver disease, severe CKD). Furthermore, in the 5 years prior to the index date, the participants without bariatric surgery had more hospitalizations and spent more days in hospital.

**Table 1:** Demographic and clinical characteristics by bariatric surgery.

Characteristics	Full cohort		
	Bariatric	No bariatric	P
N	6,212	297,945	-
Age, y	43.7 [10.5]	48.3 [16.1]	<0.001
Male	918 (14.8)	90,034 (30.2)	<0.001
Rural residence	660 (10.7)	43,163 (14.8)	<0.001
Material deprivation	3.3 [1.4]	3.3 [1.4]	0.71
Morbidities	2.3 [1.7]	1.8 [2.0]	<0.001
Sleep disturbance	843 (13.6)	8,866 (3.0)	<0.001
Hypertension	2,960 (47.6)	115,025 (38.6)	<0.001
Peptic ulcer disease	19 (0.3)	431 (0.1)	0.001
Asthma	827 (13.3)	15,131 (5.1)	<0.001
Diabetes	1,828 (29.4)	56,230 (18.9)	<0.001
Depression	1,995 (32.1)	43,538 (14.6)	<0.001
Psoriasis	94 (1.5)	2,893 (1.0)	<0.001
Hypothyroid	1,055 (17.0)	35,903 (12.1)	<0.001
Chronic pain	1,637 (26.4)	56,706 (19.0)	<0.001
Gout	445 (7.2)	23,979 (8.0)	0.01
IBS	239 (3.8)	8,238 (2.8)	<0.001
Chronic pulmonary	812 (13.1)	38,094 (12.8)	0.50
Multiple sclerosis	60 (1.0)	2,639 (0.9)	0.51
PAD	41 (0.7)	3,350 (1.1)	<0.001
Atrial fibrillation	134 (2.2)	11,733 (3.9)	<0.001
Rheumatic disease	136 (2.2)	7,025 (2.4)	0.39
Frailty	479 (7.7)	28,811 (9.7)	<0.001
Epilepsy	105 (1.7)	5,592 (1.9)	0.28
Stroke/TIA	208 (3.3)	16,148 (5.4)	<0.001
IBD	67 (1.1)	4,122 (1.4)	0.04
Severe constipation	61 (1.0)	3,762 (1.3)	0.05
Chronic heart failure	146 (2.4)	13,950 (4.7)	<0.001
Dementia	27 (0.4)	3,637 (1.2)	<0.001
Parkinson’s	10 (0.2)	1,269 (0.4)	0.002
Cancer	98 (1.6)	11,323 (3.8)	<0.001
Schizophrenia	64 (1.0)	3,977 (1.3)	0.04
Liver disease	8 (0.1)	1,084 (0.4)	0.003
CAD	65 (1.0)	11,004 (3.7)	<0.001
Alcohol misuse	81 (1.3)	9,113 (3.1)	<0.001
Severe CKD	7 (0.1)	1,698 (0.6)	<0.001
In the prior 5y			
Hospitalizations	0.6 [1.3]	0.8 [1.6]	<0.001
LOS, d	3.1 [16.4]	5.6 [30.2]	<0.001

CAD coronary artery disease, CKD chronic kidney disease, IBD inflammatory bowel disease, IBS irritable bowel syndrome, LOS length of stay, PAD peripheral artery disease, SD standard deviation, TIA transient ischemic attack

N (%) or mean (SD) as appropriate. Morbidities are sorted by the adjusted odds of receiving bariatric surgery (the adjusted odds ratios are not shown).

S2 Table gives the demographic and clinical characteristics for the sensitivity analyses. In the exact matched analysis, the remaining cohort of participants were younger, more female and had fewer morbidities. Except for age, the standardized differences of the characteristics were all less than 10% in the inverse-weight propensity scoring analyses.

### All-cause mortality

**Fig 2:**
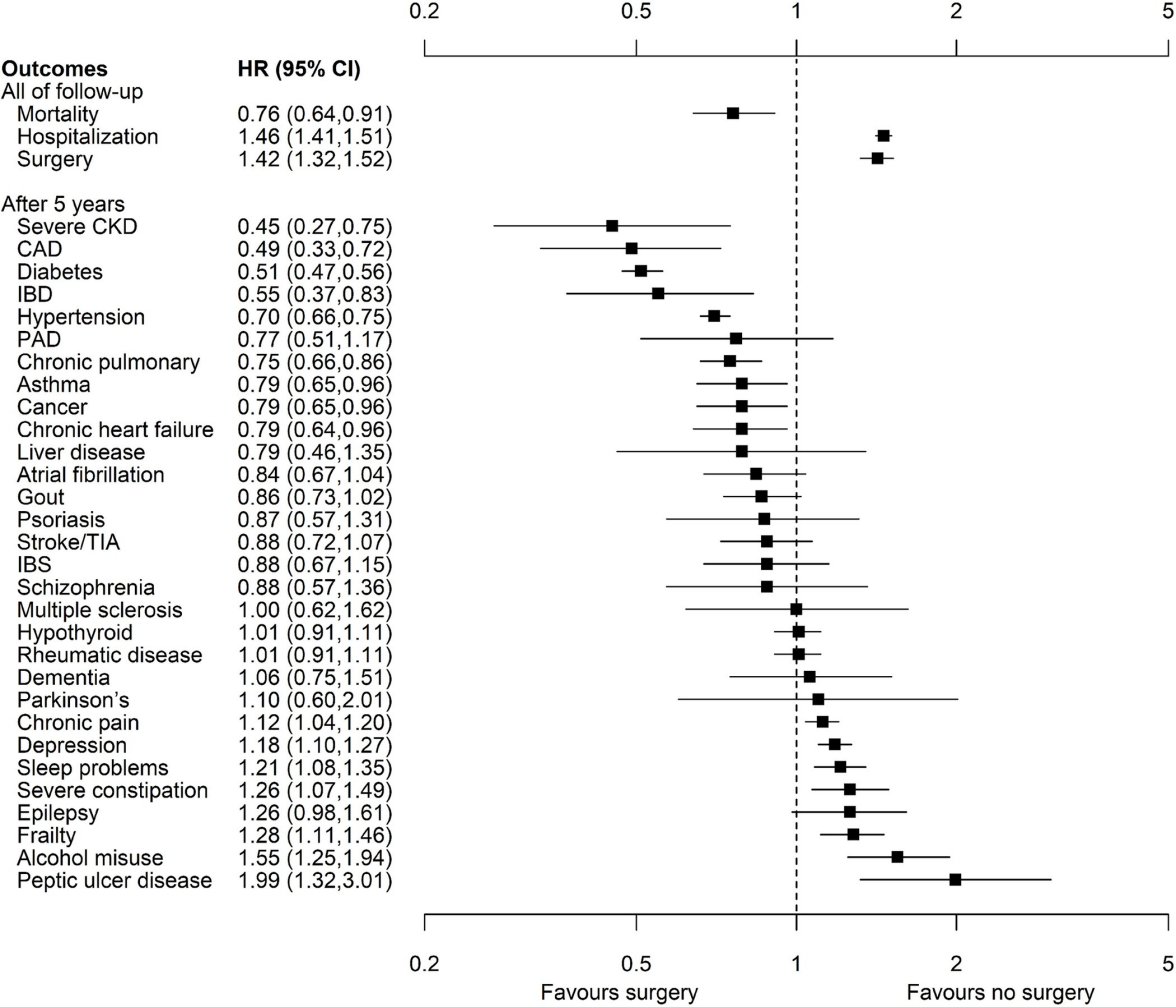
Forest plot of clinical outcomes 5–22 years after bariatric surgery. CAD coronary artery disease, CI confidence interval, CKD chronic kidney disease, HR hazards ratio, IBD inflammatory bowel disease, IBS irritable bowel syndrome, PAD peripheral artery disease, TIA transient ischemic attack.

In unadjusted analyses, bariatric surgery was associated with significantly lower mortality before and after 5 years of follow-up (HR 0.24, 95% CI 0.17,0.33, and HR 0.48, 95% CI 0.39,0.60, respectively; Table 2). With full adjustment, the risk associated with bariatric surgery remained significantly lower before 5 years of follow-up (HR 0.58, 95% CI 0.42,0.81; Fig 2), but not after 5 years of follow-up (HR 0.95, 95% CI 0.77,1.19). Over all of follow-up, bariatric surgery was associated with significantly lower mortality (HR 0.76, 95% CI 0.64,0.91).

**Table 2:** Time-to-event outcomes associated with bariatric surgery.

Outcomes	N	Events (%)	Unadjusted	Age-adjusted	Fully adjusted
Mortality					
All of follow-up	304,157	15,670 (5.2)	**0.37 (0.31,0.44)**	0.92 (0.77,1.10)	**0.76 (0.64,0.91)**
First 90 days	304,157	490 (0.2)	0.80 (0.40,1.60)	1.97 (0.97,4.00)	1.91 (0.93,3.89)
First 5 years	304,157	7,410 (2.4)	**0.24 (0.17,0.33)**	**0.68 (0.49,0.94)**	**0.58 (0.42,0.81)**
After 5 years	141,265	8,260 (5.9)	**0.48 (0.39,0.60)**	1.20 (0.97,1.50)	0.95 (0.77,1.19)
Hospitalization					
All of follow-up	304,157	140,121 (46.1)	**1.58 (1.53,1.64)**	**1.64 (1.59,1.70)**	**1.46 (1.41,1.51)**
First 90 days	304,157	12,041 (4.0)	**2.71 (2.50,2.94)**	**2.92 (2.69,3.17)**	**2.58 (2.37,2.81)**
First 5 years	304,157	104,714 (34.4)	**1.68 (1.62,1.74)**	**1.74 (1.68,1.80)**	**1.54 (1.49,1.60)**
After 5 years	141,265	65,329 (46.3)	**1.20 (1.14,1.26)**	**1.31 (1.24,1.38)**	**1.09 (1.03,1.15)**
Surgery					
All of follow-up	304,157	24,165 (7.9)	**1.79 (1.67,1.92)**	**1.61 (1.50,1.73)**	**1.42 (1.32,1.52)**
First 90 days	304,157	1,273 (0.4)	**2.89 (2.28,3.66)**	**2.68 (2.11,3.40)**	**2.23 (1.74,2.85)**
First 5 years	304,157	15,826 (5.2)	**1.76 (1.62,1.92)**	**1.61 (1.48,1.76)**	**1.44 (1.32,1.57)**
After 5 years	141,265	9,781 (6.9)	**1.87 (1.68,2.08)**	**1.63 (1.47,1.82)**	**1.38 (1.24,1.54)**
After 5 years					
Severe CKD	141,265	2,940 (2.1)	**0.24 (0.15,0.41)**	0.67 (0.40,1.12)	**0.45 (0.27,0.75)**
CAD	141,265	4,615 (3.3)	**0.26 (0.17,0.38)**	**0.40 (0.27,0.59)**	**0.49 (0.33,0.72)**
Diabetes	141,265	37,136 (26.3)	**0.71 (0.65,0.77)**	0.95 (0.88,1.04)	**0.51 (0.47,0.56)**
IBD	141,265	1,750 (1.2)	0.69 (0.47,1.03)	0.71 (0.48,1.05)	**0.55 (0.37,0.83)**
Hypertension	141,265	63,825 (45.2)	**0.68 (0.64,0.73)**	0.98 (0.92,1.04)	**0.70 (0.66,0.75)**
PAD	141,265	2,394 (1.7)	**0.46 (0.31,0.70)**	0.85 (0.57,1.29)	0.77 (0.51,1.17)
Chronic pulmonary	141,265	16,085 (11.4)	**0.67 (0.59,0.76)**	1.03 (0.90,1.18)	**0.75 (0.66,0.86)**
Asthma	141,265	3,096 (2.2)	**1.75 (1.44,2.12)**	**1.82 (1.50,2.21)**	**0.79 (0.65,0.96)**
Cancer	141,265	9,499 (6.7)	**0.54 (0.44,0.65)**	**0.79 (0.66,0.96)**	**0.79 (0.65,0.96)**
Chronic heart failure	141,265	10,841 (7.7)	**0.44 (0.36,0.54)**	1.01 (0.83,1.24)	**0.79 (0.64,0.96)**
Liver disease	141,265	1,166 (0.8)	**0.58 (0.34,0.99)**	0.77 (0.46,1.31)	0.79 (0.46,1.35)
Atrial fibrillation	141,265	8,856 (6.3)	**0.43 (0.35,0.54)**	0.92 (0.74,1.15)	0.84 (0.67,1.04)
Gout	141,265	10,725 (7.6)	**0.62 (0.53,0.73)**	**0.84 (0.71,0.996)**	0.86 (0.73,1.02)
Psoriasis	141,265	987 (0.7)	1.25 (0.84,1.86)	1.33 (0.89,1.98)	0.87 (0.57,1.31)
Stroke/TIA	141,265	7,817 (5.5)	**0.63 (0.52,0.77)**	1.04 (0.85,1.26)	0.88 (0.72,1.07)
IBS	141,265	1,878 (1.3)	**1.52 (1.16,1.97)**	**1.48 (1.14,1.93)**	0.88 (0.67,1.15)
Schizophrenia	139,752	693 (0.5)	**1.57 (1.03,2.40)**	**1.55 (1.01,2.37)**	0.88 (0.57,1.36)
Multiple sclerosis	140,261	695 (0.5)	1.27 (0.80,2.04)	**1.26 (0.79,2.02)**	1.00 (0.62,1.62)
Hypothyroid	141,265	16,183 (11.5)	**1.33 (1.21,1.47)**	**1.53 (1.39,1.69)**	1.01 (0.91,1.11)
Rheumatic disease	141,265	16,183 (11.5)	**1.33 (1.21,1.47)**	**1.53 (1.39,1.69)**	1.01 (0.91,1.11)
Dementia	140,735	3,619 (2.6)	**0.44 (0.31,0.62)**	**1.45 (1.03,2.05)**	1.06 (0.75,1.51)
Parkinson’s	140,903	775 (0.6)	0.69 (0.38,1.25)	1.37 (0.75,2.49)	1.10 (0.60,2.01)
Chronic pain	141,265	33,966 (24.0)	**1.32 (1.23,1.41)**	**1.40 (1.30,1.50)**	**1.12 (1.04,1.20)**
Depression	141,265	25,765 (18.2)	**1.81 (1.69,1.94)**	**1.77 (1.65,1.89)**	**1.18 (1.10,1.27)**
Sleep disturbance	141,265	10,896 (7.7)	**1.49 (1.34,1.67)**	**1.67 (1.49,1.87)**	**1.21 (1.08,1.35)**
Severe constipation	141,265	5,966 (4.2)	**1.27 (1.08,1.49)**	**1.78 (1.52,2.10)**	**1.26 (1.07,1.49)**
Epilepsy	141,265	2,163 (1.5)	**1.54 (1.21,1.97)**	**1.62 (1.27,2.06)**	1.26 (0.98,1.61)
Frailty	141,265	9,907 (7.0)	1.10 (0.96,1.26)	**1.61 (1.41,1.84)**	**1.28 (1.11,1.46)**
Alcohol misuse	141,265	3,043 (2.2)	**1.39 (1.12,1.72)**	**1.41 (1.14,1.75)**	**1.55 (1.25,1.94)**
Peptic ulcer disease	141,265	848 (0.6)	1.46 (0.98,2.17)	**2.14 (1.43,3.19)**	**1.99 (1.32,3.01)**

CAD coronary artery disease, CI confidence interval, HR hazard ratio, PAD peripheral artery disease, TIA transient ischemic attack

HR with 95% confidence intervals are presented.

In sensitivity analyses, the fully adjusted risks for mortality in the cohort with exact matching were not significantly different for recipients and non-recipients (S3 Table). When the alternative definition of bariatric surgery was used and with the inverse-weight propensity scoring, all results were similar to the primary analysis.

The fully adjusted risks for mortality due to external factors and mental/behavioural disorders were not significantly different for recipients versus non-recipients (full follow-up, HR 1.24, 95% CI 0.79,1.95), nor were they different for deaths to the circulatory system (HR 0.71, 95% CI 0.48,1.06). The interaction of bariatric surgery with age group (≥40 versus <40 years) was significant (P <0.001). Recipients aged ≥40 years had a lower risk of mortality (HR 0.35, 95% CI 0.28,0.43). There was no significant difference in the risk of mortality among those aged <40 years (HR 1.28, 95% CI 0.91,1.79).

When time from the index date was parametrized with restricted cubic splines using 6 knots (S2 Fig, top plot), the fully adjusted mortality risk associated with bariatric surgery was elevated until almost 1 year, then significantly decreased between 1 and 5 years, and then largely non-significantly decreased thereafter. Results were similar when we used 4 knots instead of 6 knots (S2 Fig, bottom plot).

### Hospitalization and surgery

All results from unadjusted and fully adjusted models (full follow-up, HR 1.95, 95% CI 1.89,2.01; Table 2) during the first 5 years and after 5 years (Fig 2) showed that bariatric surgery was associated with excess risk of hospitalization (Table 2). Results were similar in sensitivity analyses (S3 Table).

The hazards for time to surgery during follow-up crossed before 90 days; the magnitude of the excess risk that was associated with bariatric surgery was greater in the first 90 days but the excess risk remained throughout follow-up. Results from unadjusted, age-adjusted, and fully adjusted models (full follow-up, HR 1.42, 95% CI 1.32,1.52) demonstrated excess risk associated with bariatric surgery (Fig 2; Table 2) and were consistent in sensitivity analyses (S3 Table). Results were similar when the number of hospitalizations or days in hospital were considered instead of time to first hospitalization, or when the number of surgeries was considered instead of time to first surgery (S4 Table).

### Morbidities

After 5 years, bariatric surgery was associated with significantly lower adjusted risk of severe CKD (HR 0.45, 95% CI 0.27,0.75), CAD (HR 0.49, 95% CI 0.33,0.72), diabetes (HR 0.51, 95% CI 0.47,0.56), IBD (HR 0.55, 95% CI 0.37,0.83), hypertension (HR 0.70, 95% CI 0.66,0.75), chronic pulmonary disease (HR 0.75, 95% CI 0.66,0.86), asthma (HR 0.79, 95% 0.65,0.96), cancer (HR 0.79, 95% 0.65,0.96), and chronic heart failure (HR 0.79, 95% 0.64,0.96; Table 2; Fig 2).

In contrast, after 5 years, bariatric surgery was associated with significantly increased adjusted risk of PUD (HR 1.99, 95% CI 1.32,3.01), alcohol misuse (HR 1.55, 95% CI 1.25,1.94), frailty (HR 1.28, 95% 1.11,1.46), severe constipation (HR 1.26, 95% CI 1.07,1.49), sleep disturbance (HR 1.21, 95% CI 1.08,1.35), depression (HR 1.18, 95% CI 1.10,1.27), and chronic pain (HR 1.12, 95% CI 1.04,1.20).

Results were similar in sensitivity analyses using the alternative definition of bariatric surgery and when adjusting for the competing risk of death (S3 Table). With exact matching, bariatric surgery remained significantly associated with lower risks of only diabetes and hypertension, whereas surgery remained significantly associated with higher risks of PUD, alcohol misuse, severe constipation, sleep disturbance, depression and chronic pain. Similarly, with inverse-weight propensity scoring, bariatric surgery remained significantly associated with lower risks for some morbidities (severe CKD, CAD, diabetes, and hypertension), and higher risks with some of the other morbidities (PUD, alcohol misuse, severe constipation, sleep disturbance, depression, and chronic pain).

### Secular trends

The risk of mortality after bariatric surgery decreased over time from 1.13 (95% CI 0.85,1.50) in 2000 to 0.51 (95% CI 0.38,0.70) in 2015 (p = 0.001; S5 Table), as did the excess risk of hospitalization (from 1.63, 95% CI 1.52,1.74 in 2000, to 1.40, 95% CI 1.34,1.46 in 2015). The risk of additional surgeries did not significantly change over time.

The relative hazard of new comorbidity associated with bariatric surgery appeared stable over time for many comorbidities. However, the risks of CAD, diabetes, inflammatory bowel disease, chronic pulmonary disease and chronic heart failure, all further decreased with time (all p≤0.02) for participants with surgery versus those without, and depression and chronic pain, also, decreased with time (all p≤0.02) projecting no lower risks for participants having bariatric surgery in 2015.

### Yield of bariatric surgery

**Fig 3:**
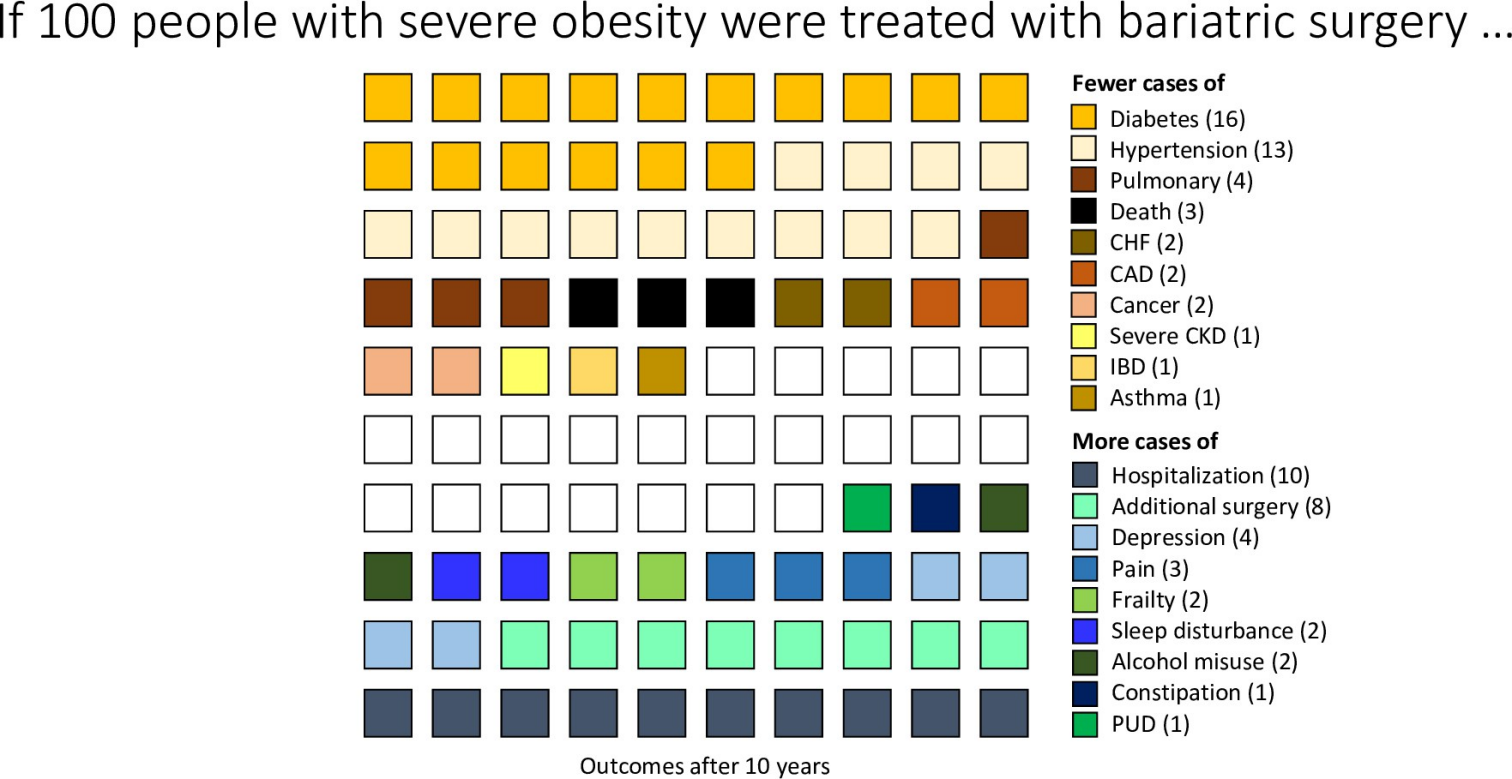
Adverse outcomes averted and experienced in 100 people 10 years after bariatric surgery. CAD coronary artery disease, CHF chronic heart failure, CKD chronic kidney disease, IBD inflammatory bowel disease, PUD peptic ulcer disease.

There were a total of 318 adverse outcomes averted per 400 recipients of bariatric surgery and a total of 125 adverse outcomes experienced per 400 recipients (Fig 3). The top 4 most frequent outcomes averted were diabetes, hypertension, severe CKD and CAD. The top 4 most frequent outcomes experienced were hospitalization, further surgery, depression and chronic pain.

The number in the brackets in the legend adjacent to the adverse outcome gives the number of outcomes averted or experienced per 100 people. Asthma was not included in chronic pulmonary disease.

## Discussion

Bariatric surgery was independently associated with a decreased risk of mortality (24%), and increased risks of hospitalization (46%) and surgery during follow-up (42%) (Fig 2). After 5 years, bariatric surgery was also associated with 11 to 55% lower risk of severe CKD, CAD, diabetes, IBD, hypertension, chronic pulmonary disease, asthma, cancer and chronic heart failure but with greater risk of multiple comorbidities (peptic ulcer, alcohol misuse, frailty, severe constipation, sleep disturbance, depression, and chronic pain), with excess risk ranging from 12% to 99%.

Previous studies examining the association between bariatric surgery and mortality have reported similar results. We found 5 randomized trials comparing various bariatric surgeries to standard of care, all in type 2 diabetic patients, with long-term mortality (mean/median follow-up of ≥5y) results (S6 Table). When results were pooled using random effects meta-analysis, the risk difference was -1.5% (95% CI -4.7,1.8) and non-significantly favoured bariatric surgery. We also found 3 meta-analyses [40–42] and a further 2 studies [43, 44] for a total of 12 individual controlled observational studies [43–54] exploring long-term mortality after bariatric surgery (S7 Table). All found that bariatric surgery was associated with significantly lower mortality (range: 16% to 64% reduction), although all were potentially vulnerable to residual confounding as none adjusted for the comprehensive panel of covariates used in the current study.

Our findings for the association between bariatric surgery and the subsequent risk of morbidities extend those from previous studies. In addition to diabetes, hypertension, CAD, chronic heart failure, and cancer, we found that the risks of severe CKD, IBD, chronic pulmonary disease, and asthma–which are all associated with hyperinsulinemia and/or inflammation–were significantly reduced, potentially by resolving prevalent cases as well as preventing incident cases. Whether some or all of these benefits are mediated through effects on hyperinsulinemia or chronic inflammation rather than weight loss will require further investigation.

We confirmed prior work showing an association between bariatric surgery and depression, alcohol misuse and fragility fractures (a component of our frailty definition). Some studies have suggested improvements in obstructive sleep apnea with bariatric surgery [55, 56]. We could not isolate OSA specifically in our dataset, but found an association between bariatric surgery and a higher risk of overall sleep disturbance. We further identified novel associations with an excess risk of peptic ulcer, severe constipation, and chronic pain. These adverse events all seem plausibly linked to bariatric surgery through effects on gastrointestinal structure or function (e.g., malabsorption; nutritional deficiency), although this explanation is speculative.

Our study has important strengths that increase confidence in its conclusions. First, results were consistent using two different algorithms for bariatric surgery and across a range of sensitivity analyses. Second, we studied a large population-based cohort of people drawn from a universal health care system. Third, we modelled mortality as a competing risk and again found generally consistent results to those in the main analysis.

Our study also has important limitations that should be considered when interpreting results. First, as with any observational study, residual confounding remains possible. We adjusted for at least twice as many variables as prior studies reporting on bariatric surgery and all-cause mortality: 4 demographic variables, 30 morbidities, and the earliest date of documented severe obesity. We also used a variety of sensitivity analyses and found generally consistent results. However, we could not adjust for potentially important confounders such as smoking status, BMI, fasting insulin or markers of inflammation (e.g., metabolic profile). In order to receive bariatric surgery in Alberta, patients must be non-smokers or have ceased smoking prior to surgery [8], which would tend to bias our results toward the alternative hypothesis, favouring bariatric surgery. In contrast, since current data show no association between BMI and mortality in general populations when models do not further adjust for fasting insulin or a marker of inflammation, lack of adjustment for BMI alone is unlikely to explain our findings [11, 12]. Second, those undergoing bariatric surgery were a small subset of the total cohort. Although the greater number of baseline morbidities in the bariatric group might suggest that these participants would have had worse prognosis with or without surgery, those in the control group had more serious morbidities and more hospitalizations in the 5 years prior to baseline, which suggests the opposite. Third, although we relied on administrative data rather than data on BMI to identify severe obesity, the results have face validity: data from Obesity Canada (obesitycanada.ca) found that 6.0% of Canadians had severe obesity in 2016 [57], which is similar to the 6.1% of adult Albertans in our dataset. Fourth, while most of the ICD-9 CCP codes used in our primary administrative algorithm identified the specific type of bariatric surgery, they were not in use until later in our follow-up timeline: sleeve gastrectomy (use of 56.93C started in 2015), Roux-en-Y gastric bypass (use of 56.93A started in 2010), and adjustable gastric banding (use of 56.93B started in 2010, use of 56.93F started in 2019). Thus, we could not explore outcomes by type of bariatric surgery, since type was often unknown and also confounded potentially by any era effect. Fifth, there may have been a few participants who were misclassified as non-recipients who had received bariatric surgery prior to April 1997 or who had surgery out-of-province, which would tend to bias against bariatric surgery.

In conclusion, bariatric surgery was associated with lower risks of certain morbidities such as diabetes, hypertension, coronary disease, severe CKD, asthma, chronic pulmonary disease, IBD, cancer and chronic heart failure as well as lower mortality. However, bariatric surgery was also associated with increased risk of hospitalization and additional surgery, as well as certain other morbidities such as alcohol misuse, osteoporosis/fragility fractures, severe constipation, peptic ulcer, sleep disturbance, depression, and chronic pain. Since values and preferences for these various benefits and harms may differ between individuals, this suggests that comprehensive counselling should be offered to patients considering bariatric surgery to help them explore this tradeoff (Fig 3), especially since many patients lose <50% of excess weight postoperatively [15]. Future longitudinal studies should continue to monitor the effects of bariatric surgery on wider panels of comorbidities and outcomes, and investigate how to improve bariatric surgical techniques and associated postoperative care.

## Supporting information

S1 Checklist STROBE statement—checklist of items that should be included in reports of *cohort studies*. (DOCX)

S2 Checklist Human participants research checklist. (DOCX)

S1 Fig Quantile-quantile plot for randomly sampled index dates for non-recipients. The distribution of the index dates for the non-recipients is similar to the distribution of the index dates for the recipients.
(PDF)

S2 Fig Restricted cubic spline for all-cause mortality: Bariatric surgery versus no surgery. The top plot shows a spline for bariatric surgery versus no bariatric surgery with 6 knots: 90 days, 1, 5, 10, 15, and 20 years. The bottom plot shows a spline with 4 knots: 1, 5, 10, and 15 years. The histogram shows the percentage of participants available at each year after bariatric surgery. Splines allow the relative mortality risk to be flexibly modelled over time. The intervals between the knots were restricted to cubic polynomials or polynomials of a lesser degree. The first and the last intervals were forced to be linear polynomials. The top plot places a knot at 90 days, and shows a non-significant high risk for mortality immediately after bariatric surgery. In the bottom plot, the knot at 90 days is removed, which smooths estimates of risk over the first year, and shows an overall lower risk in mortality in the first year after surgery. After the first year, the mortality risk gradually climbs to the line of unity after 15 years, with a non-significantly higher risk after 20 years.
(PDF)

S1 Table Administrative algorithms for bariatric surgeries. BMI body mass index, GI gastrointestinal.
(PDF)

S2 Table Demographic and clinical characteristics by bariatric surgery–sensitivity analyses. CAD coronary artery disease, CKD chronic kidney disease, IBD inflammatory bowel disease, IBS irritable bowel syndrome, PAD peripheral artery disease, PUD peptic ulcer disease, SD standard deviation, TIA transient ischemic attack. (%) or mean (SD) as appropriate. Morbidities are sorted by the adjusted odds of receiving bariatric surgery (the adjusted odds ratios are not shown). The standardized differences are all less than 10% in the inverse-weight propensity scoring analysis except for age.
(PDF)

S3 Table Time-to-event outcomes associated with bariatric surgery–sensitivity analyses. (PDF)

S4 Table Count outcomes associated with bariatric surgery. CI confidence interval, RR rate ratio RR with 95% confidence intervals are presented.
(PDF)

S5 Table Time-to-event outcomes associated with bariatric surgery by fiscal year of bariatric surgery. CAD coronary artery disease, CI confidence interval, CKD chronic kidney disease, HR hazard ratio HR with 95% confidence intervals are presented.
(PDF)

S6 Table Long-term all-cause mortality in randomized trials. There were 4 deaths in 412 participants. When results were pooled using random effects meta-analysis, the risk difference was -1.5% (95% CI -4.7,1.8) and non-significantly favoured bariatric surgery. The risk ratio was 0.46 (95% 0.16,1.35) after adding 1 to each cell (due to the high frequency of 0 cells) also non-significantly favoured bariatric surgery.
(PDF)

S7 Table Long-term all-cause mortality in controlled observational studies. BMI body mass index, BP blood pressure, CAD coronary artery disease, CI confidence interval, ESKD end-stage kidney disease, GERD gastroesophageal reflux disease, HR hazards ratio, HTN hypertension, MI myocardial infarction, OR odds ratio, RR risk ratio, SBP systolic blood pressure. ^**1**^Calculated using methods by Woods *et al*. [58].
(PDF)
