# Supplementary material for: Long-term clinical outcomes of bariatric surgery in adults with severe obesity: A population-based retrospective cohort study
Source: PLoS One. 2024 Jun 6;19(6):e0298402. doi: 10.1371/journal.pone.0298402 (PMC11156280; doi:10.1371/journal.pone.0298402)
Supplement: S1 Fig — The distribution of the index dates for the non-recipients is similar to the distribution of the index dates for the recipients. (PDF) [file pone.0298402.s003.pdf]

S1 Fig. Quantile-quantile plot for randomly sampled index dates for non-recipients

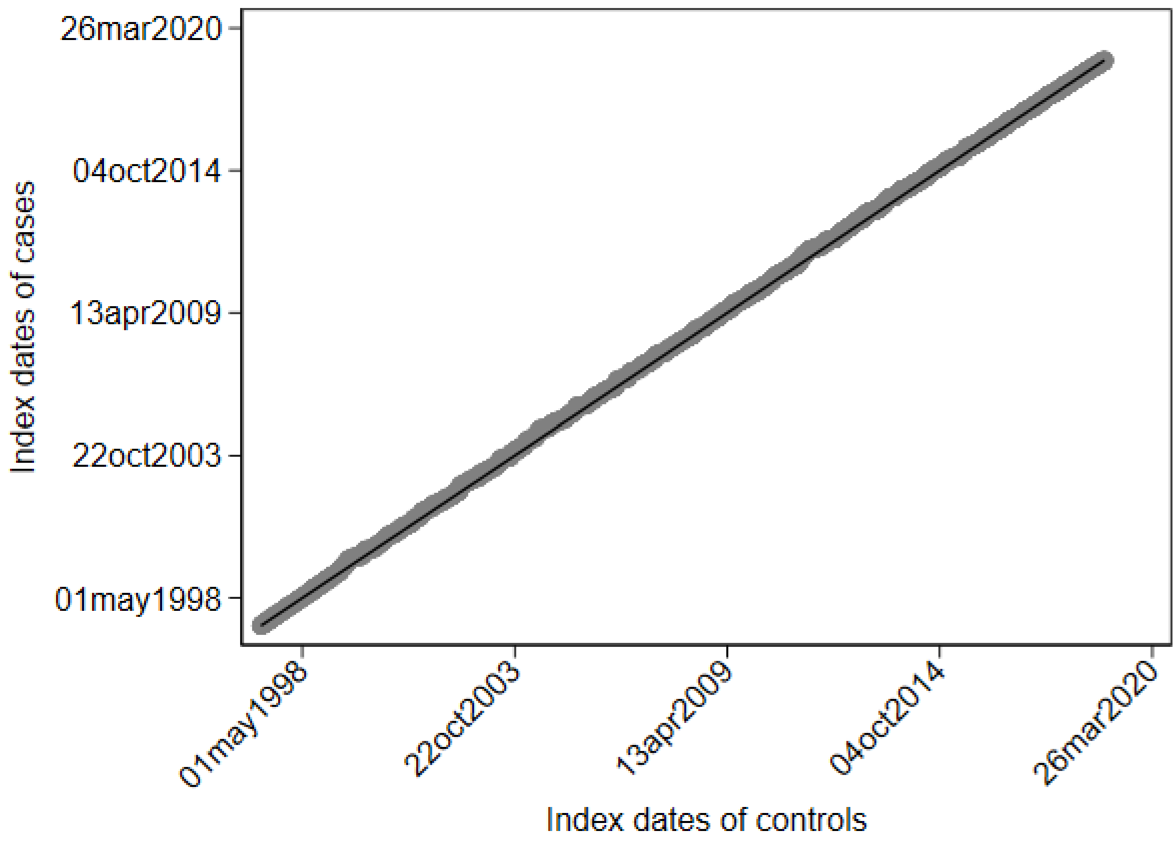

The distribution of the index dates for the non-recipients is similar to the distribution of the index dates for the recipients.
