# Supplementary material for: Long-term clinical outcomes of bariatric surgery in adults with severe obesity: A population-based retrospective cohort study
Source: PLoS One. 2024 Jun 6;19(6):e0298402. doi: 10.1371/journal.pone.0298402 (PMC11156280; doi:10.1371/journal.pone.0298402)
Supplement: S2 Fig — The top plot shows a spline for bariatric surgery versus no bariatric surgery with 6 knots: 90 days, 1, 5, 10, 15, and 20 years. The bottom plot shows a spline with 4 knots: 1, 5, 10, and 15 years. The histogram shows the percentage of participants available at each year after bariatric surgery. Splines allow the relative mortality risk to be flexibly modelled over time. The intervals between the knots were restricted to cubic polynomials or polynomials of a lesser degree. The first and the last intervals were forced to be linear polynomials. The top plot places a knot at 90 days, and shows a non-significant high risk for mortality immediately after bariatric surgery. In the bottom plot, the knot at 90 days is removed, which smooths estimates of risk over the first year, and shows an overall lower risk in mortality in the first year after surgery. After the first year, the mortality risk gradually climbs to the line of unity after 15 years, with a non-significantly higher risk after 20 years. (PDF) [file pone.0298402.s004.pdf]

S2 Fig. Restricted cubic spline for all-cause mortality: bariatric surgery versus no surgery

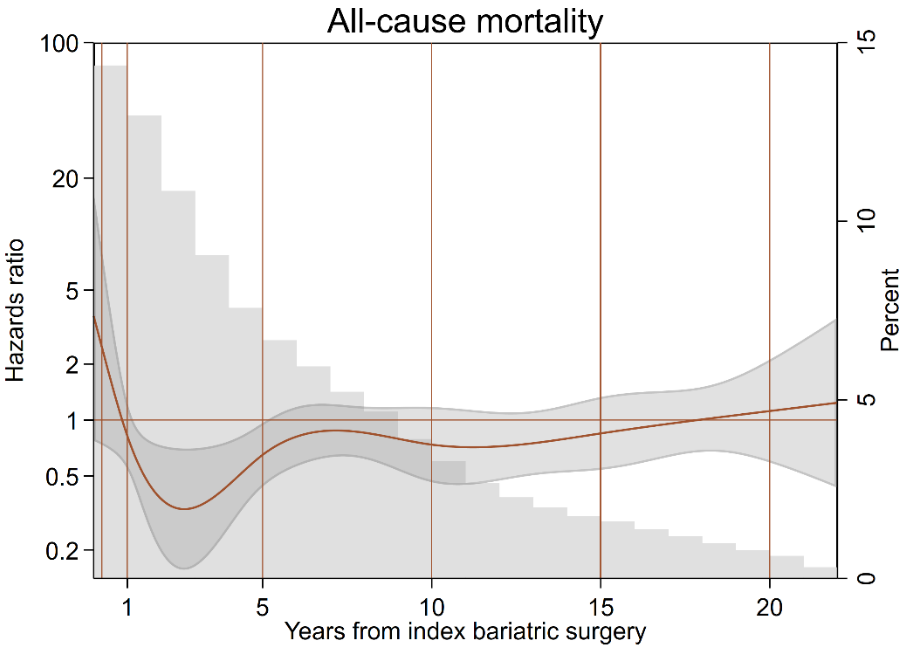

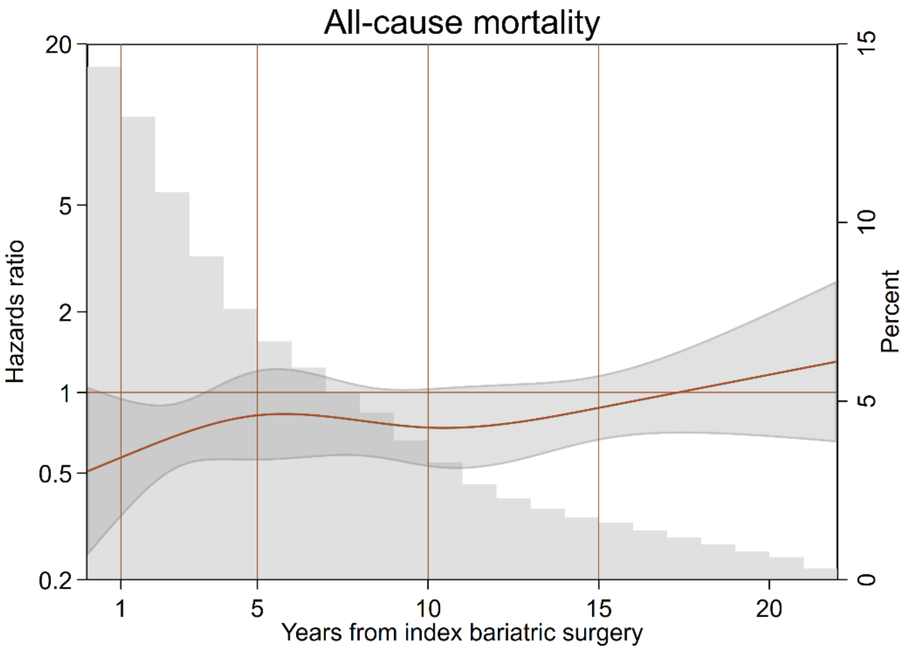

The top plot shows a spline for bariatric surgery versus no bariatric surgery with 6 knots: 90 days, 1, 5, 10, 15, and 20 years. The bottom plot shows a spline with 4 knots: 1, 5, 10, and 15 years. The histogram shows the percentage of participants available at each year after bariatric surgery. Splines allow the relative mortality risk to be flexibly modelled over time. The intervals between the knots were restricted

to cubic polynomials or polynomials of a lesser degree. The first and the last intervals were forced to be linear polynomials.

The top plot places a knot at 90 days, and shows a non-significant high risk for mortality immediately after bariatric surgery. In the bottom plot, the knot at 90 days is removed, which smooths estimates of risk over the first year, and shows an overall lower risk in mortality in the first year after surgery. After the first year, the mortality risk gradually climbs to the line of unity after 15 years, with a non-significantly higher risk after 20 years.
