# Supplementary material for: Long-term clinical outcomes of bariatric surgery in adults with severe obesity: A population-based retrospective cohort study
Source: PLoS One. 2024 Jun 6;19(6):e0298402. doi: 10.1371/journal.pone.0298402 (PMC11156280; doi:10.1371/journal.pone.0298402)
Supplement: S1 Table — BMI body mass index, GI gastrointestinal. (PDF) [file pone.0298402.s005.pdf]

S1 Table. Administrative algorithms for bariatric surgeries

Algorithm	Bariatric surgery	Obesity	Non-bariatric gastrointestinal surgeries
Bariatric surgery (1 claim) **and**<br>obesity (1 BMI modifier) **and no**<br>non-bariatric GI surgeries (1 hospitalization)	ICD-9 CCP:<br>56.93<br>RYGB 56.93A<br>Band 56.93B, D, E, F<br>Sleeve 56.93C	BMI modifier	ICD-9-CM diagnosis:<br>150-159 (GI cancer)<br>230 (abdominal cancer)<br>531-533 (perforated GI ulcer)<br><br>ICD-10-CA diagnosis:<br>C15 (GI cancer)<br>D05 (abdominal cancer)<br>K25 K26 K27 (perforated GI ulcer)
Bariatric surgery (1 hospitalization) **and**<br>obesity (BMI modifier) **and no**<br>non-bariatric GI surgeries (1 hospitalization)	ICD-9-CM procedure:<br>43.89, 44.39<br><br>ICD-10-CA CCI:<br>1NF78	BMI modifier	ICD-9-CM diagnosis:<br>150-159 (GI cancer)<br>230 (abdominal cancer)<br>531-533 (perforated GI ulcer)<br><br>ICD-10-CA diagnosis:<br>C15 (GI cancer)<br>D05 (abdominal cancer)<br>K25 K26 K27 (perforated GI ulcer)

BMI body mass index, GI gastrointestinal
