# Supplementary material for: Long-term clinical outcomes of bariatric surgery in adults with severe obesity: A population-based retrospective cohort study
Source: PLoS One. 2024 Jun 6;19(6):e0298402. doi: 10.1371/journal.pone.0298402 (PMC11156280; doi:10.1371/journal.pone.0298402)
Supplement: S2 Table — CAD coronary artery disease, CKD chronic kidney disease, IBD inflammatory bowel disease, IBS irritable bowel syndrome, PAD peripheral artery disease, PUD peptic ulcer disease, SD standard deviation, TIA transient ischemic attack. (%) or mean (SD) as appropriate. Morbidities are sorted by the adjusted odds of receiving bariatric surgery (the adjusted odds ratios are not shown). The standardized differences are all less than 10% in the inverse-weight propensity scoring analysis except for age. (PDF) [file pone.0298402.s006.pdf]

S2 Table. Demographic and clinical characteristics by bariatric surgery – sensitivity analyses

Characteristics	Full cohort			Inverse-weight propensity scoring			Exact matches	
	Bariatric	No bariatric	Standardized difference, %	Bariatric	No bariatric	Standardized difference, %	Bariatric	No bariatric
N	6,212	297,945	-	6,212	297,945	-	3,241	62,371
Age, y	43.7 [10.5]	48.3 [16.1]	-33.9	46.2 [10.6]	48.2 [16.0]	-15.0	41.6 [10.2]	41.6 [10.3]
Male	918 (14.8)	90,034 (30.2)	-37.6	(27.1)	(29.9)	-6.3	364 (11.2)	7,005 (11.2)
Rural residence	660 (10.7)	43,163 (14.8)	-34.3	(15.7)	(14.4)	-3.6	221 (6.9)	4,253 (6.9)
Material deprivation	3.3 [1.4]	3.3 [1.4]	-6.3	3.5 [1.7]	3.5 [1.8]	0.6	3.3 [1.3]	3.3 [1.3]
Morbidities	2.3 [1.7]	1.8 [2.0]	27.8	2.0 [1.7]	2.0 [2.0]	1.4	1.3 [1.0]	1.3 [1.0]
Obesity date	-	-	-21.6	-	-	5.5	-	-
Sleep disturbance	843 (13.6)	8,866 (3.0)	39.2	(3.6)	(3.2)	2.1	72 (2.2)	1,386 (2.2)
Hypertension	2,960 (47.6)	115,025 (38.6)	18.3	(34.5)	(38.8)	-9.0	1,154 (35.6)	22,208 (35.6)
PUD	19 (0.3)	431 (0.1)	3.4	(0.1)	(0.1)	-1.4	0 (0.0)	0 (0.0)
Asthma	827 (13.3)	15,131 (5.1)	28.8	(5.7)	(5.3)	2.0	141 (4.4)	2,713 (4.4)
Diabetes	1,828 (29.4)	56,230 (18.9)	24.8	(17.4)	(19.1)	-4.5	582 (18.0)	11,200 (18.0)
Depression	1,995 (32.1)	43,538 (14.6)	42.3	(16.7)	(15.0)	4.7	738 (22.8)	14,202 (22.8)
Psoriasis	94 (1.5)	2,893 (1.0)	4.9	(0.8)	(1.0)	-2.3	5 (0.2)	96 (0.2)
Hypothyroid	1,055 (17.0)	35,903 (12.1)	14.0	(12.5)	(12.2)	1.0	339 (10.5)	6,524 (10.5)
Chronic pain	1,637 (26.4)	56,706 (19.0)	17.5	(20.9)	(19.2)	4.2	603 (18.6)	11,604 (18.6)
Gout	445 (7.2)	23,979 (8.0)	-3.3	(7.7)	(8.0)	-1.3	85 (2.6)	1,636 (2.6)
IBS	239 (3.8)	8,238 (2.8)	6.1	(3.4)	(2.8)	3.4	26 (0.8)	500 (0.8)
Chronic pulmonary	812 (13.1)	38,094 (12.8)	0.9	(13.6)	(12.8)	2.4	164 (5.1)	3,156 (5.1)
Multiple sclerosis	60 (1.0)	2,639 (0.9)	0.8	(1.2)	(0.9)	2.8	4 (0.1)	77 (0.1)
PAD	41 (0.7)	3,350 (1.1)	-4.9	(0.8)	(1.1)	-2.8	1 (0.0)	19 (0.0)
Atrial fibrillation	134 (2.2)	11,733 (3.9)	-10.4	(3.8)	(3.9)	-0.4	8 (0.2)	154 (0.2)
Rheumatic disease	136 (2.2)	7,025 (2.4)	-1.1	(2.2)	(2.4)	-0.9	3 (0.1)	58 (0.1)
Frailty	479 (7.7)	28,811 (9.7)	-7.0	(11.0)	(9.6)	4.5	76 (2.3)	1,463 (2.3)
Epilepsy	105 (1.7)	5,592 (1.9)	-1.4	(2.4)	(1.9)	3.4	2 (0.1)	38 (0.1)
Stroke/TIA	208 (3.3)	16,148 (5.4)	-10.1	(5.1)	(5.4)	-1.4	18 (0.6)	346 (0.6)
IBD	67 (1.1)	4,122 (1.4)	-2.8	(1.5)	(1.4)	1.4	4 (0.1)	77 (0.1)
Severe constipation	61 (1.0)	3,762 (1.3)	-2.7	(1.4)	(1.3)	1.4	3 (0.1)	58 (0.1)
Chronic heart failure	146 (2.4)	13,950 (4.7)	-12.7	(4.9)	(4.6)	1.1	3 (0.1)	58 (0.1)
Dementia	27 (0.4)	3,637 (1.2)	-8.7	(3.9)	(3.8)	-5.0	0 (0.0)	0 (0.0)
Parkinson’s	10 (0.2)	1,269 (0.4)	-4.9	(0.7)	(1.2)	-2.9	0 (0.0)	0 (0.0)
Cancer	98 (1.6)	11,323 (3.8)	-13.8	(0.3)	(0.4)	0.8	15 (0.5)	289 (0.5)
Schizophrenia	64 (1.0)	3,977 (1.3)	-2.8	(2.0)	(1.3)	5.0	3 (0.1)	58 (0.1)
Liver disease	8 (0.1)	1,084 (0.4)	-4.7	(0.2)	(0.4)	-2.4	0 (0.0)	0 (0.0)

Characteristics	Full cohort			Inverse-weight propensity scoring			Exact matches	
	Bariatric	No bariatric	Standardized difference, %	Bariatric	No bariatric	Standardized difference, %	Bariatric	No bariatric
CAD	65 (1.0)	11,004 (3.7)	-17.5	(3.6)	(3.6)	-0.2	3 (0.1)	58 (0.1)
Alcohol misuse	81 (1.3)	9,113 (3.1)	-12.0	(4.9)	(3.0)	9.4	7 (0.2)	135 (0.2)
Severe CKD	7 (0.1)	1,698 (0.6)	-7.8	(0.2)	(0.6)	-6.2	0 (0.0)	0 (0.0)

CAD coronary artery disease, CKD chronic kidney disease, IBD inflammatory bowel disease, IBS irritable bowel syndrome, PAD peripheral artery disease, PUD peptic ulcer disease, SD standard deviation, TIA transient ischemic attack

(%) or mean (SD) as appropriate. Morbidities are sorted by the adjusted odds of receiving bariatric surgery (the adjusted odds ratios are not shown).

The standardized differences are all less than 10% in the inverse-weight propensity scoring analysis except for age. The cohort with exact matches includes only 21.6% of the full cohort.
