# Supplementary material for: Long-term clinical outcomes of bariatric surgery in adults with severe obesity: A population-based retrospective cohort study
Source: PLoS One. 2024 Jun 6;19(6):e0298402. doi: 10.1371/journal.pone.0298402 (PMC11156280; doi:10.1371/journal.pone.0298402)
Supplement: S3 Table — (PDF) [file pone.0298402.s007.pdf]

S3 Table. Time-to-event outcomes associated with bariatric surgery – sensitivity analyses

Outcomes	N	Fully adjusted	CIHI definition	Death as a competing risk	Inverse-weight propensity scoring	N	Many to many exact matching
Mortality							
All of follow-up	304,157	**0.76 (0.64,0.91)**	**0.79 (0.66,0.95)**	-	**0.58 (0.43,0.78)**	65,612	0.81 (0.59,1.12)
First 5 years	304,157	**0.58 (0.42,0.81)**	**0.76 (0.58,0.99)**	-	**0.40 (0.26,0.61)**	65,612	0.64 (0.32,1.27)
After 5 years	141,265	0.95 (0.77,1.19)	0.87 (0.67,1.13)	-	0.85 (0.47,1.51)	22,318	1.09 (0.61,1.95)
Hospitalization							
All of follow-up	304,157	**1.46 (1.41,1.51)**	**1.41 (1.36,1.46)**	**-**	**1.34 (1.26,1.41)**	65,612	**1.75 (1.65,1.86)**
First 5 years	304,157	**1.54 (1.49,1.60)**	**1.45 (1.40,1.51)**	-	**1.44 (1.36,1.54)**	65,612	**1.88 (1.77,2.01)**
After 5 years	141,265	**1.09 (1.03,1.15)**	**1.09 (1.03,1.16)**	**1.15 (1.09,1.21)**	1.05 (0.95,1.16)	22,318	**1.31 (1.19,1.45)**
Surgery							
All of follow-up	304,157	**1.42 (1.32,1.52)**	**1.38 (1.28,1.49)**	**-**	**1.38 (1.25,1.51)**	65,612	**1.52 (1.37,1.69)**
First 5 years	304,157	**1.44 (1.32,1.57)**	**1.46 (1.34,1.60)**	**-**	**1.34 (1.20,1.51)**	65,612	**1.39 (1.22,1.59)**
After 5 years	141,265	**1.38 (1.24,1.54)**	**1.22 (1.07,1.40)**	**1.42 (1.27,1.59)**	**1.48 (1.22,1.79)**	22,318	**1.58 (1.33,1.87)**
After 5 years							
Severe CKD	141,265	**0.45 (0.27,0.75)**	**0.55 (0.33,0.91)**	**0.44 (0.27,0.74)**	**0.28 (0.14,0.60)**	22,318	0.38 (0.13,1.13)
CAD	141,265	**0.49 (0.33,0.72)**	**0.31 (0.17,0.56)**	**0.51 (0.34,0.76)**	**0.38 (0.21,0.69)**	22,318	0.57 (0.30,1.09)
Diabetes	141,265	**0.51 (0.47,0.56)**	**0.45 (0.41,0.49)**	**0.61 (0.57,0.66)**	**0.54 (0.46,0.64)**	22,318	**0.53 (0.45,0.62)**
IBD	141,265	**0.55 (0.37,0.83)**	**0.55 (0.34,0.88)**	**0.59 (0.39,0.88)**	0.71 (0.35,1.45)	22,318	0.56 (0.24,1.32)
Hypertension	141,265	**0.70 (0.66,0.75)**	**0.61 (0.56,0.65)**	**0.77 (0.73,0.82)**	**0.80 (0.71,0.92)**	22,318	**0.65 (0.58,0.73)**
Chronic pulmonary	141,265	**0.75 (0.66,0.86)**	**0.75 (0.65,0.88)**	**0.80 (0.70,0.92)**	0.81 (0.62,1.06)	22,318	0.85 (0.66,1.11)
Asthma	141,265	**0.79 (0.65,0.96)**	**0.66 (0.51,0.86)**	0.85 (0.69,1.03)	0.93 (0.68,1.28)	22,318	0.94 (0.62,1.43)
Cancer	141,265	**0.79 (0.65,0.96)**	**0.76 (0.61,0.96)**	**0.81 (0.67,0.98)**	0.71 (0.49,1.04)	22,318	0.85 (0.63,1.16)
Chronic heart failure	141,265	**0.79 (0.64,0.96)**	**0.75 (0.60,0.95)**	**0.80 (0.65,0.97)**	0.74 (0.54,1.03)	22,318	0.92 (0.61,1.37)
Chronic pain	141,265	**1.12 (1.04,1.20)**	**1.13 (1.04,1.23)**	**1.18 (1.10,1.26)**	**1.17 (1.04,1.33)**	22,318	**1.16 (1.02,1.31)**
Depression	141,265	**1.18 (1.10,1.27)**	**1.19 (1.09,1.29)**	**1.24 (1.15,1.33)**	**1.46 (1.29,1.66)**	22,318	**1.34 (1.18,1.51)**

Outcomes	N	Fully adjusted	CIHI definition	Death as a competing risk	Inverse-weight propensity scoring	N	Many to many exact matching
Sleep disturbance	141,265	**1.21 (1.08,1.35)**	**1.24 (1.08,1.41)**	**1.28 (1.14,1.43)**	**1.37 (1.14,1.65)**	22,318	**1.39 (1.13,1.70)**
Severe constipation	141,265	**1.26 (1.07,1.49)**	**1.37 (1.13,1.66)**	**1.33 (1.12,1.56)**	1.27 (0.92,1.75)	22,318	**1.42 (1.01,1.99)**
Frailty	141,265	**1.28 (1.11,1.46)**	**1.30 (1.11,1.53)**	**1.33 (1.16,1.52)**	**1.53 (1.13,2.06)**	22,318	1.18 (0.92,1.52)
Alcohol misuse	141,265	**1.55 (1.25,1.94)**	**1.96 (1.56,2.46)**	**1.63 (1.30,2.03)**	1.18 (0.81,1.73)	22,318	**2.45 (1.64,3.65)**
Peptic ulcer disease	141,265	**1.99 (1.32,3.01)**	**1.83 (1.10,3.07)**	**2.09 (1.39,3.16)**	2.73 (0.82,9.04)	22,318	**3.11 (1.35,7.22)**

CAD coronary artery disease, CI confidence interval, HR hazard ratio, PAD peripheral artery disease, SHR subdistribution hazard ratio, TIA transient ischemic attack

HR or SHR with 95% confidence intervals are presented.
