# Supplementary material for: Long-term clinical outcomes of bariatric surgery in adults with severe obesity: A population-based retrospective cohort study
Source: PLoS One. 2024 Jun 6;19(6):e0298402. doi: 10.1371/journal.pone.0298402 (PMC11156280; doi:10.1371/journal.pone.0298402)
Supplement: S4 Table — CI confidence interval, RR rate ratio RR with 95% confidence intervals are presented. (PDF) [file pone.0298402.s008.pdf]

S4 Table. Count outcomes associated with bariatric surgery

Outcomes	Events (rate /1000 pty)	Unadjusted	Age-adjusted	Fully adjusted
Hospitalizations				
All of follow-up	363,744 (185)	**1.25 (1.20,1.29)**	**1.42 (1.37,1.47)**	**1.28 (1.24,1.32)**
First 5 years	195,263 (181)	**1.40 (1.34,1.47)**	**1.57 (1.50,1.64)**	**1.42 (1.36,1.47)**
After 5 years	168,481 (106)	**1.09 (1.03,1.15)**	**1.23 (1.17,1.30)**	**1.09 (1.04,1.15)**
Days in hospital				
All of follow-up	3,350,852 (1,705)	**0.66 (0.62,0.71)**	**1.42 (1.37,1.47)**	**1.16 (1.09,1.23)**
First 5 years	1,681,760 (1,556)	**0.68 (0.63,0.74)**	**1.18 (1.09,1.27)**	**1.22 (1.13,1.31)**
After 5 years	1,669,092 (1,049)	**0.80 (0.73,0.89)**	**1.25 (1.14,1.38)**	1.08 (0.99,1.19)
Surgeries				
All of follow-up	29,062 (15)	**1.79 (1.65,1.94)**	**1.61 (1.49,1.75)**	**1.42 (1.32,1.54)**
First 5 years	17,477 (16)	**1.75 (1.59,1.91)**	**1.60 (1.46,1.75)**	**1.43 (1.30,1.56)**
After 5 years	11,585 (7)	**1.79 (1.60,2.01)**	**1.59 (1.42,1.77)**	**1.45 (1.30,1.63)**

CI confidence interval, RR rate ratio

RR with 95% confidence intervals are presented.
