# Supplementary material for: Long-term clinical outcomes of bariatric surgery in adults with severe obesity: A population-based retrospective cohort study
Source: PLoS One. 2024 Jun 6;19(6):e0298402. doi: 10.1371/journal.pone.0298402 (PMC11156280; doi:10.1371/journal.pone.0298402)
Supplement: S5 Table — CAD coronary artery disease, CI confidence interval, CKD chronic kidney disease, HR hazard ratio HR with 95% confidence intervals are presented. (PDF) [file pone.0298402.s009.pdf]

S5 Table. Time-to-event outcomes associated with bariatric surgery by fiscal year of bariatric surgery

Outcomes	Fully adjusted	Fiscal year 2000	Fiscal year 2005	Fiscal year 2010	Fiscal year 2015	P-value
Mortality						
All of follow-up	**0.76 (0.64,0.91)**	1.13 (0.85,1.50)	0.87 (0.72,1.05)	**0.67 (0.54,0.82)**	**0.51 (0.38,0.70)**	0.001
First 5 years	**0.58 (0.42,0.81)**	0.96 (0.33,2.79)	0.78 (0.39,1.58)	**0.64 (0.43,0.95)**	**0.52 (0.36,0.74)**	0.30
After 5 years	0.95 (0.77,1.19)	1.11 (0.04,1.48)	0.90 (0.72,1.12)	0.73 (0.52,1.02)	**0.59 (0.35,0.996)**	0.06
Hospitalization						
All of follow-up	**1.46 (1.41,1.51)**	**1.63 (1.52,1.74)**	**1.55 (1.48,1.62)**	**1.47 (1.42,1.52)**	**1.40 (1.34,1.46)**	<0.001
First 5 years	**1.54 (1.49,1.60)**	**2.08 (1.92,2.25)**	**1.83 (1.73,1.93)**	**1.61 (1.55,1.67)**	**1.42 (1.36,1.48)**	<0.001
After 5 years	**1.09 (1.03,1.15)**	**1.24 (1.14,1.34)**	**1.11 (1.06,1.18)**	1.00 (0.93,1.07)	0.90 (0.81,1.01)	<0.001
Surgery						
All of follow-up	**1.42 (1.32,1.52)**	**1.46 (1.27,1.67)**	**1.48 (1.35,1.62)**	**1.50 (1.40,1.61)**	**1.52 (1.39,1.67)**	0.60
First 5 years	**1.44 (1.32,1.57)**	**1.33 (1.04,1.70)**	**1.40 (1.19,1.65)**	**1.48 (1.34,1.64)**	**1.56 (1.42,1.72)**	0.23
After 5 years	**1.38 (1.24,1.54)**	**1.63 (1.39,1.90)**	**1.48 (1.33,1.66)**	**1.36 (1.17,1.58)**	1.24 (0.98,1.57)	0.10
After 5 years						
Severe CKD	**0.45 (0.27,0.75)**	0.49 (0.23,1.05)	**0.44 (0.26,0.73)**	**0.39 (0.19,0.78)**	0.34 (0.11,1.05)	0.64
CAD	**0.49 (0.33,0.72)**	0.71 (0.47,1.08)	**0.34 (0.20,0.59)**	**0.16 (0.06,0.43)**	**0.08 (0.02,0.33)**	0.004
Diabetes	**0.51 (0.47,0.56)**	**0.58 (0.51,0.67)**	**0.53 (0.48,0.58)**	**0.47 (0.43,0.52)**	**0.43 (0.36,0.50)**	0.01
IBD	**0.55 (0.37,0.83)**	0.95 (0.56,1.62)	**0.56 (0.37,0.84)**	**0.33 (0.17,0.63)**	**0.19 (0.07,0.54)**	0.02
Hypertension	**0.70 (0.66,0.75)**	**0.71 (0.64,0.78)**	**0.70 (0.65,0.75)**	**0.69 (0.64,0.75)**	**0.68 (0.60,0.77)**	0.66
Chronic pulmonary	**0.75 (0.66,0.86)**	0.95 (0.80,1.12)	**0.73 (0.63,0.84)**	**0.56 (0.45,0.69)**	**0.43 (0.31,0.60)**	<0.001
Asthma	**0.79 (0.65,0.96)**	**0.77 (0.60,0.99)**	**0.78 (0.63,0.95)**	0.78 (0.57,1.07)	0.79 (0.49,1.28)	0.94
Cancer	**0.79 (0.65,0.96)**	0.81 (0.63,1.05)	**0.78 (0.65,0.95)**	0.76 (0.57,1.01)	**0.73 (0.47,1.14)**	0.71
Chronic heart failure	**0.79 (0.64,0.96)**	1.10 (0.86,1.40)	**0.74 (0.60,0.91)**	**0.50 (0.36,0.69)**	**0.34 (0.20,0.57)**	<0.001
Chronic pain	**1.12 (1.04,1.20)**	**1.20 (1.09,1.31)**	**1.11 (1.03,1.19)**	1.02 (0.92,1.14)	0.94 (0.80,1.11)	0.02
Depression	**1.18 (1.10,1.27)**	**1.35 (1.23,1.49)**	**1.20 (1.11,1.29)**	1.06 (0.95,1.17)	0.94 (0.80,1.10)	0.001
Sleep disturbance	**1.21 (1.08,1.35)**	**1.23 (1.05,1.44)**	**1.22 (1.09,1.38)**	**1.21 (1.03,1.42)**	1.20 (0.94,1.54)	0.88
Severe constipation	**1.26 (1.07,1.49)**	**1.28 (1.02,1.60)**	**1.25 (1.06,1.48)**	1.23 (0.97,1.56)	1.21 (0.84,1.76)	0.83
Frailty	**1.28 (1.11,1.46)**	1.18 (0.98,1.42)	**1.28 (1.12,1.47)**	**1.40 (1.15,1.70)**	**1.53 (1.13,2.07)**	0.21
Alcohol misuse	**1.55 (1.25,1.94)**	**1.50 (1.09,2.07)**	**1.56 (1.25,1.95)**	**1.63 (1.21,2.19)**	**1.69 (1.06,2.70)**	0.72

Outcomes	Fully adjusted	Fiscal year 2000	Fiscal year 2005	Fiscal year 2010	Fiscal year 2015	P-value
Peptic ulcer disease	**1.99 (1.32,3.01)**	**2.05 (1.23,3.42)**	**1.98 (1.30,3.02)**	1.91 (0.99,3.67)	1.84 (0.68,5.03)	0.87

CAD coronary artery disease, CI confidence interval, CKD chronic kidney disease, HR hazard ratio

HR with 95% confidence intervals are presented.
