# Supplementary material for: Long-term clinical outcomes of bariatric surgery in adults with severe obesity: A population-based retrospective cohort study
Source: PLoS One. 2024 Jun 6;19(6):e0298402. doi: 10.1371/journal.pone.0298402 (PMC11156280; doi:10.1371/journal.pone.0298402)
Supplement: S6 Table — There were 4 deaths in 412 participants. When results were pooled using random effects meta-analysis, the risk difference was -1.5% (95% CI -4.7,1.8) and non-significantly favoured bariatric surgery. The risk ratio was 0.46 (95% 0.16,1.35) after adding 1 to each cell (due to the high frequency of 0 cells) also non-significantly favoured bariatric surgery. (PDF) [file pone.0298402.s010.pdf]

S6 Table. Long-term all-cause mortality in randomized trials

Study and funding sources	Surgery era	Recipients	Non-recipients	Follow-up	Deaths
Mingrone 2021 [1]<br><br>Foundation	2009-2011	40	20 (excluding 2 withdrawals, including 2 crossovers)	10y	0 deaths in the recipients and 1 death in the non-recipients
Courcoulas 2020 [2]<br><br>Public	2009-2011	41 (excluding 4 withdrawals)	20 (excluding 3 withdrawals)	5y	0 deaths in either group
Ikramuddin 2018 [3]<br><br>Private, public	2008-2011	55 (excluding 5 withdrawals, including potentially 2 crossovers)	43 (excluding 17 withdrawals, including potentially 9 crossovers)	5y	0 deaths in the recipients and 1 death in the non-recipients
Schauer 2017 [4]<br><br>Private, public	2007-2011	99 (excluding 4 withdrawal)	42 (excluding 11 withdrawals, including 1 crossover)	5y	0 deaths in the recipients and 1 death in the non-recipients
O'Brien 2013 [5]<br><br>Private, public	2000-2001	28 (excluding 7 withdrawals)	24 (excluding 16 withdrawals, including 17 crossovers)	10y	1 death in the recipients and 0 deaths in the non-recipients

There were 4 deaths in 412 participants. When results were pooled using random effects meta-analysis, the risk difference was -1.5% (95% CI -4.7,1.8) and non-significantly favoured bariatric surgery. The risk ratio was 0.46 (95% 0.16,1.35) after adding 1 to each cell (due to the high frequency of 0 cells) also non-significantly favoured bariatric surgery.

References

1. Mingrone G, Panunzi S, De Gaetano A, Guidone C, Iaconelli A, Capristo E, et al. Metabolic surgery versus conventional medical therapy in patients with type 2 diabetes: 10-year follow-up of an open-label, single-centre, randomised controlled trial. Lancet. 2021;397(10271):293-304. Epub 2021/01/25. doi: 10.1016/S0140-6736(20)32649-0.
2. Courcoulas AP, Gallagher JW, Neiberg RH, Eagleton EB, DeLany JP, Lang W, et al. Bariatric Surgery vs Lifestyle Intervention for Diabetes Treatment: 5-Year Outcomes From a Randomized Trial. J Clin Endocrinol Metab. 2020;105(3):866-76. Epub 2020/01/10. doi: 10.1210/clinem/dgaa006.
3. Ikramuddin S, Korner J, Lee WJ, Thomas AJ, Connett JE, Bantle JP, et al. Lifestyle Intervention and Medical Management With vs Without Roux-en-Y Gastric Bypass and Control of Hemoglobin A1c, LDL Cholesterol, and Systolic Blood Pressure at 5 Years in the Diabetes Surgery Study. JAMA. 2018;319(3):266-78. Epub 2018/01/18. doi: 10.1001/jama.2017.20813.
4. Schauer PR, Bhatt DL, Kirwan JP, Wolski K, Aminian A, Brethauer SA, et al. Bariatric Surgery versus Intensive Medical Therapy for Diabetes - 5-Year Outcomes. N Engl J Med. 2017;376(7):641-51. Epub 2017/02/16. doi: 10.1056/NEJMoa1600869.
5. O'Brien PE, Brennan L, Laurie C, Brown W. Intensive medical weight loss or laparoscopic adjustable gastric banding in the treatment of mild to moderate obesity: long-term follow-up of a prospective randomised trial. Obes Surg. 2013;23(9):1345-53. Epub 2013/06/14. doi: 10.1007/s11695-013-0990-3.
