# Supplementary material for: Long-term clinical outcomes of bariatric surgery in adults with severe obesity: A population-based retrospective cohort study
Source: PLoS One. 2024 Jun 6;19(6):e0298402. doi: 10.1371/journal.pone.0298402 (PMC11156280; doi:10.1371/journal.pone.0298402)
Supplement: S7 Table — BMI body mass index, BP blood pressure, CAD coronary artery disease, CI confidence interval, ESKD end-stage kidney disease, GERD gastroesophageal reflux disease, HR hazards ratio, HTN hypertension, MI myocardial infarction, OR odds ratio, RR risk ratio, SBP systolic blood pressure. 1Calculated using methods by Woods et al. [58]. (PDF) [file pone.0298402.s011.pdf]

S7 Table. Long-term all-cause mortality in controlled observational studies

Study and funding sources	Surgery era	Recipients	Non-recipients	Follow-up	HR (95% CI)	Methods to account for confounding
Adams 2023 [1]<br><br>Private, public, foundation	2010-2018	21,837<br>(3 large bariatric surgery registries, health facility records, or the Enterprise Data Warehouses)	21,837<br>(Utah driver license records or ID cards)	Mean 13.2y	0.84 (0.79,0.90)	Matched 1:1 on age, sex, BMI, surgery/driver license application year
Carlsson 2020 [2] (prospective)<br><br>Private, public, foundation	1987-2001	2,007<br>(Swedish obesity study)	2,040<br>(Swedish obesity study matched non-recipients)<br><br>1,135<br>(Swedish obesity study reference non-recipients)	Median 23.0y	0.70 (0.61,0.81) Matched non-recipients<br><br>1.84 (1.49,2.27)[1] Unmatched reference non-recipients	Matched 1:1 on age, sex, weight, height, waist and hip circumference, SBP, cholesterol, triglycerides, smoking status, diabetes, menopause, 4 psychosocial variables, 2 personality traits and adjusted for age, sex, education, marriage/partner status, smoking status, year of inclusion, BMI, waist:hip ratio, cardiovascular disease, glucose tolerance, hypertension, fasting insulin, cholesterol, substance abuse, psychiatric care or psychiatric drug use
Moussa 2020 [3]	1987-2019	3,701	3,701	Mean 11.7y	0.25 (0.18,0.35)	Matched 1:1 on age, sex, BMI and adjusted for smoking status, HTN, hyperlipidemia, diabetes, alcohol use,

Study and funding sources	Surgery era	Recipients	Non-recipients	Follow-up	HR (95% CI)	Methods to account for confounding
Public, foundation		(Clinical Practice Research Datalink)	(Clinical Practice Research Datalink)			cocaine use, exercise, medications (BB, CCB, ACE-i or ARBs, statins, aspirin, HRT)
Sheetz 2020 [4] (ESKD only)<br><br>Not reported	2006-2015	1,597 (USRDS)	4,750 (USRDS)	5y	0.69 (0.60,0.78)	Matched 1:1-3 on age, sex, ethnicity, BMI, census region, initiation of dialysis, Mahalanobis distance (based on age, smoking status, type 2 diabetes, coronary artery disease, peripheral vascular disease, chronic obstructive pulmonary disease, stroke/TIA, hypertension, heart failure)
Ceriani 2019 [5]<br><br>Public, Internal	1999-2008	472 (bariatric clinics)	1,405 (bariatric clinics – declined surgery)	Mean 12.1y	0.53 (0.29,0.97)	Matched 1:3-4 on age, sex, BMI, BP, diabetes and adjusted for age, sex, BMI, BP, diabetes, coronary heart disease, eGFR
Thereaux 2019 [6]<br><br>None	2009	8,966 (Système National des Données de Santé)	8,966 (Système National des Données de Santé)	Mean 6.8y	Gastric bypass 0.64 (0.52,0.78)<br>Sleeve gastrectomy 0.38 (0.29,0.50)	Matched 1:1 on age, sex, BMI, hypoglycemics and adjusted for age, sex, and BMI plus other unknown statistically significant variables
Pontiroli 2018 [7]<br><br>Public, Internal	1995-2001	385 (bariatric clinics)	681 (bariatric clinics – declined surgery)	Mean 19.5y	0.52 (0.33,0.80)	Matched n:n on age, sex, BMI, BP, diabetes
Lent 2017 [8]<br><br>Public	2004-2015	2,428 (medical center)	2,428 (primary care clinic)	Median 6.5y	0.65 (0.50,0.84)	Matched 1:1 on age, sex, BMI, diabetes and adjusted for smoking status, statins, antihypertensives

Study and funding sources	Surgery era	Recipients	Non-recipients	Follow-up	HR (95% CI)	Methods to account for confounding
Davidson 2016 [9]<br><br>Private, Public	1984-2002	7,925 (bariatric clinic)	7,925 (Utah driver license and identification card applicants)	Mean 7.2y	0.60 (0.50,0.73)	Matched 1:1 on age, sex, BMI, surgery/driver license application year and adjusted for age, sex, BMI (cubic term)
Arterburn 2015 [10]<br><br>Public	2000-2011	2,500 (Veterans Affairs)	7,462 (Veterans Affairs)	Mean 6.7y	5-14y<br>0.47 (0.39,0.58)	Matched 1:1-3 on age, sex, BMI, ethnicity, geographic region, diabetes, Mahalanobis distance (age, BMI, diagnostic cost group)
Guidry 2015 [11]<br><br>Public	2002-2003	401 (tertiary care center)	401 (tertiary care center)	Median 11.9y	OR<br>0.48 (0.29,0.78)	Propensity score matched 1:1 on age, sex, ethnicity, diabetes, other endocrine, neurologic, pulmonary, HTN, hyperlipidemia, CAD, MI, valvular, aneurysmal, GERD, hematologic, cancer, psychiatric or substance misuse, insurance
Busetto 2007 [12]<br><br>Not reported	1994-2001	821 (bariatric clinic)	821 (epidemiologic survey at 6 tertiary obesity clinics)	Mean 6.4y	0.36 (0.16,0.79)	Matched 1:1 on age, sex, BMI and adjusted for age, sex, BMI

BMI body mass index, BP blood pressure, CAD coronary artery disease, CI confidence interval, ESKD end-stage kidney disease, GERD gastroesophageal reflux disease, HR hazards ratio, HTN hypertension, MI myocardial infarction, OR odds ratio, RR risk ratio, SBP systolic blood pressure

[1]Calculated using methods by Woods *et al*.

References

1. Adams TD, Meeks H, Fraser A, Davidson LE, Holmen J, Newman M, et al. Long-term all-cause and cause-specific mortality for four bariatric surgery procedures. Obesity. 2023;31:574-85.
2. Carlsson LMS, Sjoholm K, Jacobson P, Andersson-Assarsson JC, Svensson P, Taube M, et al. Life Expectancy after Bariatric Surgery in the Swedish Obese Subjects Study. N Engl J Med. 2020;383:1535-43.
3. Moussa O, Ardissino M, Heaton T, Tang A, Khan O, Ziprin P, et al. Effect of bariatric surgery on long-term cardiovascular outcomes: a nationwide nested cohort study. Obesity and Metabolic Syndrome. 2020;41:2660-7.
4. Sheetz KH, Gerhardinger L, Dimick JB, Waits SA. Bariatric Surgery and Long-term Survival in Patients With Obesity and End-stage Kidney Disease. JAMA Surg. 2020;155(7):581-8. Epub 2020/05/28. doi: 10.1001/jamasurg.2020.0829.
5. Ceriani V, Sarro G, Micheletto G, Glovanelli A, Zakaria AS, Fanchini M, et al. Long-term mortality in obese subjects undergoing malabsorptive surgery (biliopancreatic diversion and biliointestinal bypass) versus medical treatment. Int J Obes. 2019;43:1147-53.
6. Thereaux J, Lesuffleur T, Czernichow S, Basevant A, Msika S, Nocca D, et al. Long-term adverse events after sleeve gastrectomy or gastric bypass: a 7-year nationwide, observational, population-based, cohort study. Lancet Diabetes Endocrinology. 2019;7:786-95.
7. Pontiroli AE, Zakaria AS, Fanchini M, Oslo C, Tagliabue E, Micheletto G, et al. A 23-year study of mortality and development of co-morbidities in patients with obesity undergoing bariatric surgery (laparoscopic gastric banding) in comparison with medical treatment of obesity. Cardiovasc Diabetol. 2018;17:161.
8. Lent MR, Benotti PN, Mirshahi T, Gerhard GS, Strodel WE, Petrick AT, et al. All-Cause and Specific-Cause Mortality Risk After Roux-en-Y Gastric Bypass in Patients With and Without Diabetes. Diabetes Care. 2017;40:1379-85.
9. Davidson LE, Adams TD, Kim J, Jones JL, Hashibe M, Taylor D, et al. Association of Patient Age at Gastric Bypass Surgery With Long-term All-Cause and Cause-Specific Mortality. JAMA Surg. 2016;151(7):631-7. doi: 10.1001/jamasurg.2015.5501.
10. Arterburn DE, Olsen MK, Smith VA, Livingston EH, Van Scoyoc L, Yancy WS, Jr., et al. Association between bariatric surgery and long-term survival. JAMA. 2015;313(1):62-70. doi: 10.1001/jama.2014.16968.
11. Guidry CA, Davies SW, Sawyer RG, Schirmer BD, Hallowell PT. Gastric bypass improves survival compared with propensity-matched controls: a cohort study with over 10-year follow-up. Am J Surg. 2015;209(3):463-7. Epub 20141223. doi: 10.1016/j.amjsurg.2014.10.009.
12. Busetto L, Mirabelli D, Petroni ML, Mazza M, Favretti F, Segato G, et al. Comparative long-term mortality after laparoscopic adjustable gastric banding versus nonsurgical controls. Surg Obes Relat Dis. 2007;3(5):496-502; discussion doi: 10.1016/j.soard.2007.06.003.
